# Anterior Clinoidal Meningiomas: Meningeal Anatomical Considerations and Surgical Implications

**DOI:** 10.3389/fonc.2020.00634

**Published:** 2020-05-25

**Authors:** Tao Xu, Yong Yan, Alexander I. Evins, Zhenyu Gong, Lei Jiang, Huaiyu Sun, Li Cai, Hongxiang Wang, Weiqing Li, Yicheng Lu, Ming Zhang, Juxiang Chen

**Affiliations:** ^1^Department of Neurosurgery, Changzheng Hospital, Naval Medical University, Shanghai, China; ^2^Department of Neurological Surgery, Weill Cornell Medicine, New York Presbyterian Hospital, New York, NY, United States; ^3^Department of Neurosurgery, Tiemei General Hospital, Liaoning, China; ^4^Arkansas Neuroscience Institute, St. Vincent Hospital, Little Rock, AR, United States; ^5^Department of Pathology, Changzheng Hospital, Naval Medical University, Shanghai, China; ^6^Department of Anatomy, University of Otago, Dunedin, New Zealand

**Keywords:** anterior clinoidal meningioma, meninges, classification, surgical anatomy, cavernous sinus, carotid artery, anterior clinoid process

## Abstract

**Objective:** Surgical removal of anterior clinoidal meningiomas (ACMs) remains a challenge because of its complicated relationship with surrounding meninges, major arteries and cranial nerves. This study aims to define the meningeal structures around the anterior clinoid process (ACP) and its surgical implications.

**Methods:** Five dry skulls and 19 cadavers were used in the anatomical study. Cadavers were prepared as transverse, coronal, and sagittal plastinated sections, and the meningeal architecture around the ACP was studied with dissecting and confocal microscopies. The database of meningiomas in one single center was retrospectively reviewed, and the patients with ACMs were collected for clinical analysis.

**Results:** The superior, lateral, medial surfaces, and the tip of ACP were covered by different layers and types of meninges. The ACMs were classified into four main types based on the sites of origin, possible extending pathways following meningeal dura. In the retrospective cohort of 131 ACMs, the percentage of types I, IIa, IIb, III, and IV were 42.0% (55/131), 19.8% (26/131), 9.2% (12/131), 16.8% (22/131), and 12.2% (16/131), respectively. We found that types IIa and I had higher chances for achieving Simpson grade 1–2 resection (92.3 and 85.4%, respectively), followed by type III (54.5%) and type IV (31.3%), while type IIb showed little chance of Simpson grade 1–2 resection. Univariate and multivariate analyses revealed ACM classification and tumor size (<3 cm) to be independent risk factors for achieving more extensive resection.

**Conclusion:** The meningeal architecture around the ACP may guide and determine the origin and extension of ACMs. The classification based on the meningeal architecture helps to understand surgical anatomy as well as predicting surgical outcomes.

## Introduction

Anterior clinoidal meningiomas (ACMs) were first reported in 1938 and compose about 34.0–43.9% of all sphenoid wing meningiomas (1). ACMs originate from the meninges attached to the anterior clinoidal process (ACP) and extend along the meningeal dura as they grow larger, displacing or even invading the surrounding neurovascular structures (2). The close relationship between the tumor and critical structures may result in high surgical morbidity and recurrence rate (2–5).

Because of the complex regional meningeal anatomy, meningiomas originated from different areas of ACP may present with varying patterns of growth and surgical outcomes. There were several anatomical studies on the ACP and its surrounding structures, especially the cavernous sinus and the carotid artery (6–9). However, few reports revealed the fine architecture of the meningeal coverings of the ACP, and few of the previously proposed classifications of ACMs differentiated the underlying relationship between the meningeal architecture and surgical implications.

In this study, we used some new anatomical techniques to investigate the fine meningeal architecture around the ACP, proposed a new classification based on anatomical findings, and analyzed the characteristics of different types of ACMs in 131 cases.

## Materials and Methods

### Cadaveric Study

Five dry skulls and 19 cadavers (8 female and 11 male; age range, 54–89 years; mean age, 75 years) were used in this anatomical study. Written informed consent from the donor or the next of kin was obtained under the Human Tissues Act.

The dry skulls were used for studying the structural characteristics. The cadavers were prepared for 19 sets of plastinated sections. The thickness of the section was about 2.5–3.0 mm. Sheet plastination is a modern anatomical technique in which water and lipids of tissues and cells are replaced by curable and transparent resin. The plastination procedure was performed as previously described (10). The prepared section was examined under a Leica MZ8 stereoscopic dissecting microscope (Leica, Heerbrugg, Switzerland). The high-resolution images of the selected areas were scanned and collected with an Epson Perfection V750 Pro Scanner (Epson, Jakarta, Indonesia), in which the scanning resolution was set up at 1,200–6,400 dpi. The plastination process results in collagen fibers and neurofilaments being endogenously autofluorescent at the 488-nm excitation (11). Differentiation among those autofluorescent fibers is based upon their morphology, fluorescent intensity, and anatomical distribution. The plastinated section was observed under a Nikon AIR confocal laser scanning microscope (Nikon, Tokyo, Japan). The thickness of the optical section was set up at 16.7 μm under a 10 × objective, and the images were electronically recorded and montaged.

### Clinical Study

The tumor registration database of Changzheng Hospital, Naval Medical University, Shanghai, China, was retrospectively reviewed. The clinical and pathological characteristics were extracted from the database and charts. The inclusion criteria were as follows: patients received surgery for removing the meningiomas and the diagnosis confirmed by magnetic resonance imaging (MRI), intraoperative findings, and pathological reports. The primary outcomes included the extent of tumor removal, cranial nerve function, and surgical-related morbidities. The extent of resection (Simpson grade) was recorded in the operative notes according to the chief surgeon's impression under the microscope, as well as confirmed by postoperative imaging study. Postoperatively, all patients underwent a brain computed tomography (CT) or MRI within 2 days after surgery. Follow-up was done with clinical and imaging examination of the patients. Two researchers (TX and YY) collected the data as well as classified the tumors with guidance from senior professors (YL and JC); any divergence was discussed and resolved.

### Statistical Analysis

The clinical data were recorded and analyzed by SPSS 25.0 (Chicago, IL, USA). The univariate and multivariate analyses were performed using the logistic regression; *p* < 0.05 was regarded as statistically significant.

## Results

### Zoning of the Anterior Clinoid Process

**Figure 1:**
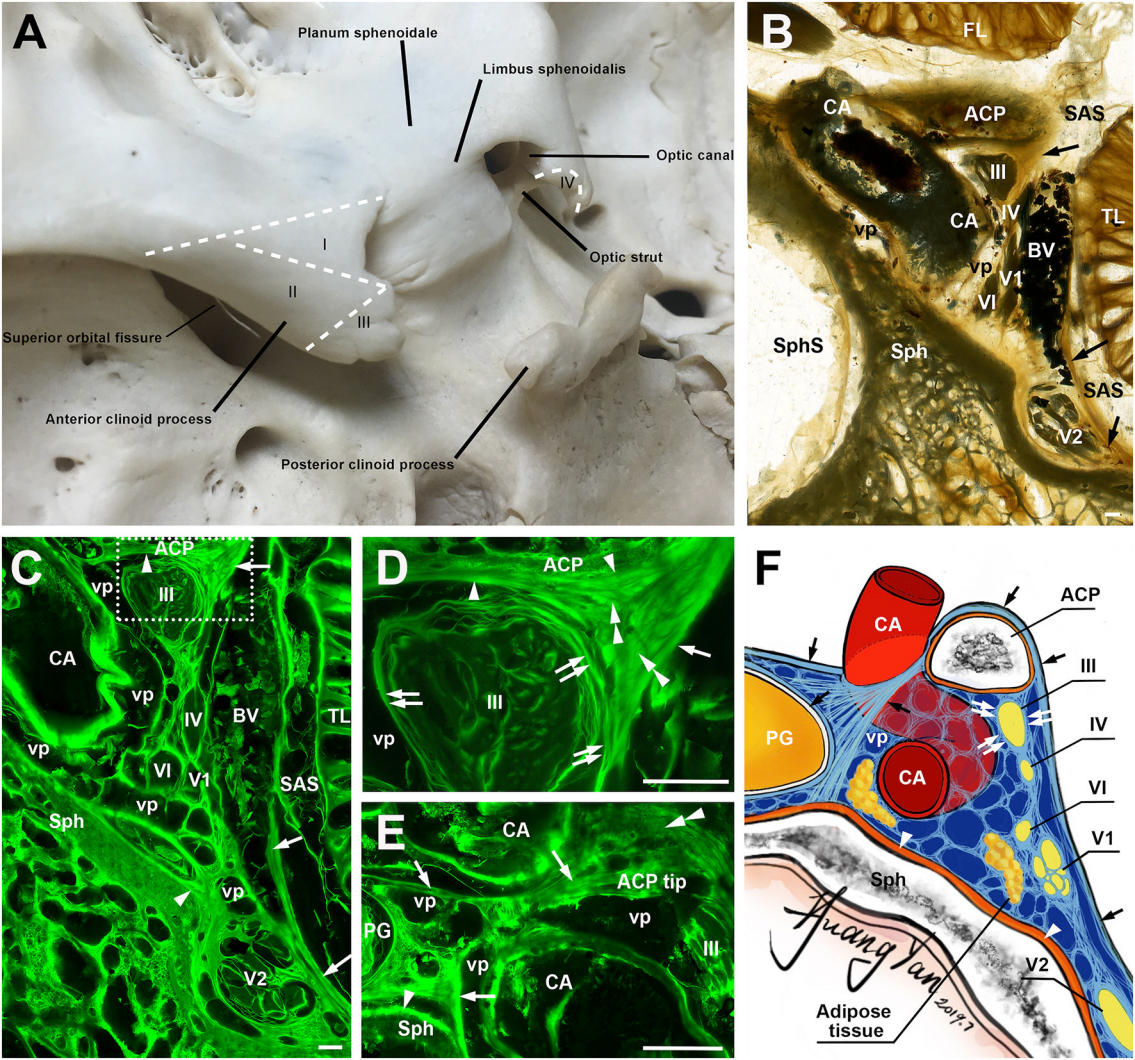
The anterior clinoid process (ACP) and its meningeal coverings. **(A)** A posterosuperior view of the ACP, showing its four zones (I–IV). **(B)** A coronal sheet plastination section at the level of the clinoidal segment of the carotid artery (CA). Arrows point the lateral wall of cavernous sinus. **(C)** is the mirror confocal image of **(B)**. Arrowheads point to the periosteal dura. Arrows point to the meningeal dura. **(D)** is the higher magnification of the dashed line box of **(C)**, showing the meningeal architecture (single arrow and arrowheads) on the inferolateral surface of the ACP and its relationship with the dural sleeves (double arrows) of the ocular motor (III) and the lateral wall of the cavernous sinus. Double arrowheads point to sagittally orientated meningeal dural fibers, which originated from the tentorium and inserted to the ACP. **(E)** is from an adjacent section through the tip of the ACP, 3.4 mm posterior to **(D)**, showing the meningeal dural fibrous bundles (arrows) between C4 and C6 segments of the CA. **(F)** An illustration showing the meningeal architecture of the ACP and its surrounding structures that were mentioned in the previous images. BV, cerebral bridging vein; vp, venous plexus; Sph, sphenoid bone; SphS, sphenoid sinus; TL, temporal lobe; FL, frontal lobe; SAS, subarachnoid space; cranial nerves II, III, IV, V_1_, V_2_, and VI; bars = 1 mm.

The ACP was the posteromedial extremity of the sphenoid ridge and appeared like an irregular triangular mass. It is located between the superior orbital fissure laterally and the optic canal medially. Based on these fixed bony landmarks, we could further divide ACP into four areas (Figure 1A). Following the lesser sphenoid wing, there is a ridge that separates the ACP into areas I and II. Area I was the superior surface of the ACP that was almost flat and continued with the planum sphenoidale and optic canal. Area II was the lateral surface that gradually curved down and then turned medially. Area III was the triangle posterior to the end of the ridge (tip of ACP). The interclinoid dural fold and the anterior petroclinoid dural fold were attached to the tip. Area IV was the medial surface of ACP that was adjacent to the optic canal anteromedially and the internal carotid artery (ICA) posteromedially. The inferior surface of ACP faced the clinoidal ICA and was not visible during surgery unless a total removal of ACP.

### The Meningeal Architecture of the Anterior Clinoid Process

The meningeal architecture around the ACP varied (Figures 1B–E). Area I of the ACP was covered by the arachnoid and both the meningeal and periosteal dura (Figure 1B). The covering of area II of the ACP was complicated, as a multiple layered meningeal dura was sandwiched in between the arachnoid and periosteal dura (Figures 1C,D). The fiber orientations of these meningeal dura layers were various, but most of them were sagittal along a posteroanterior axis and inserted to the ACP. The superficial (or lateral) layer continued with the meningeal dura on the anterior and middle cranial fossae, the roof and lateral wall of the cavernous sinus (Figure 1C). The deeper (or medial) layers contributed to the meningeal dural sleeves of the ocular motor and trochlear nerves (Figures 1C,D) and the fibrous mesh network within the cavernous sinus.

The meninges covering area III include both periosteal and meningeal dura continued from areas I and II, mixed with fibers from the tentorium, which inserted into the tip of the ACP and serviced as the demarcation between the inferomedial and inferolateral surfaces of the ACP (Figure 1E). The oculomotor nerve pieced the single meningeal dura of the oculomotor triangle (roof of the cavernous sinus) and entered into the cavernous sinus just posteriorly to the ACP.

The meningeal dura of area I extended medially, forming the meningeal dural fibrous bundles (distal dural ring) between C4 and C6 segments of the CA, attached and fused with the external membrane (tunica adventitia) of the ICA (Figure 1E). Above this dural ring, the superior part of area IV was also covered by both the meningeal and periosteal dura, while the inferior surface of area IV was covered only by the periosteal dura (Figures 1D,E). The above findings are illustrated in Figure 1F.

### Classification of ACMs and Its Surgical Relevance

**Figure 2:**
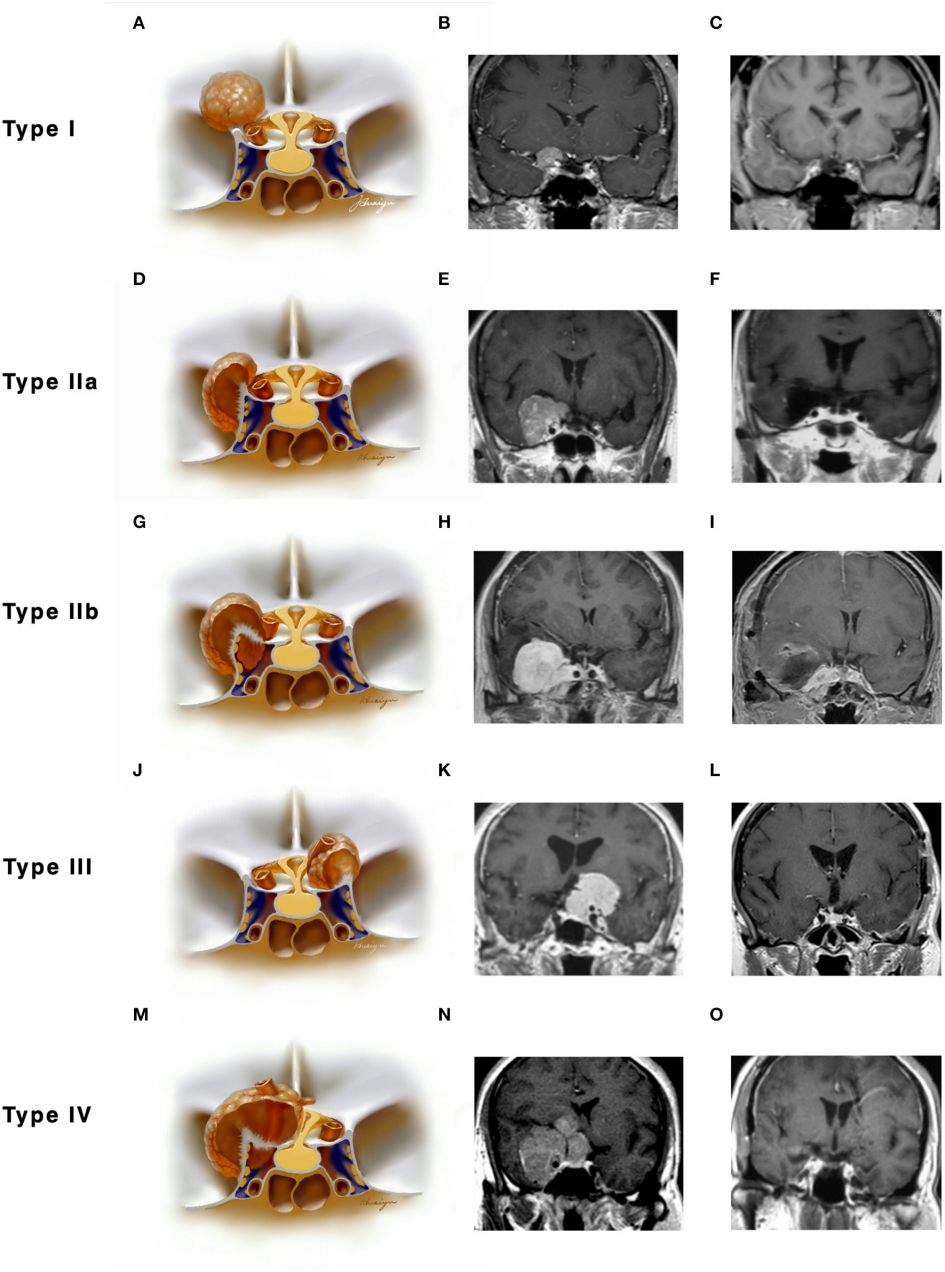
Classifications of anterior clinoidal meningiomas (ACMs) based on meningeal anatomy of the anterior clinoid process (ACP). Each horizontal panel represents an illustration of coronal sectional view, a preoperative and postoperative MR images of a type of ACMs**. (A–C)** Type I ACMs originate from the superior surface of the ACP. Note that the tumor may invade into the optic canal following the falciform ligament. **(D–F)** Type IIa ACMs originate from the lateral surface of ACP. They “attach” and “push” rather than invade the cavernous sinus because of the thick and multilayer meningeal on the lateral wall of cavernous sinus. **(G–I)** Type IIb ACMs originate from the tip of the ACP and grow both inside and outside of the cavernous sinus following the meningeal dura near the oculomotor triangle. **(J–L)** Type III ACMs that originated from the medial surface of ACP; they could affect the distal dural ring and wrap the ICA from the very beginning. **(M–O)** Type IV ACMs that extend to multiple directions following the meninges.

We classified the ACMs into four types based on the location of their origin, meningeal architecture, extending pattern, as well as surgical implications (Table 1, Figure 2).

**Table 1:** Classification of anterior clinoidal meningiomas and surgical implications.

**Tumor classification**	**Common origin**	**Extending pattern**	**Recommended surgical techniques**
Type I 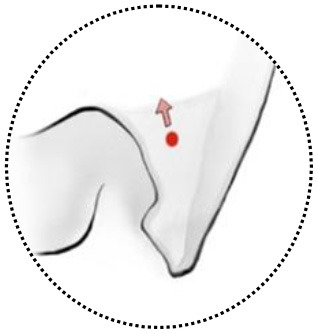	Superior surface of the ACP	Grow superiorly and laterally to the supraclinoidal space;	Frontal-temporal craniotomy +/– Anterior clinoidectomy +/– Unroof the optic canal
Type IIa 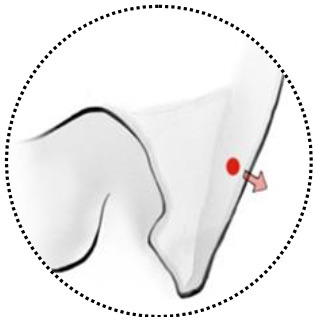	Lateral surface of the ACP	Grow along the lateral wall of cavernous sinus;	Frontal-temporal craniotomy+ Zygomatic osteotomy+/– Anterior clinoidectomy +/– Unroof the superior orbital fissure
Type IIb 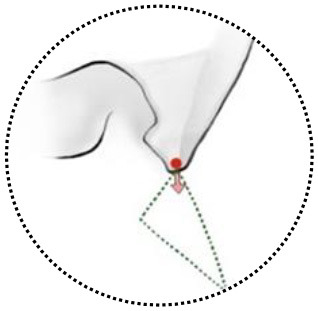	Tip of the ACP	Grow both inside and outside of the cavernous sinus following the meningeal dura near the oculomotor triangle;	Frontal-temporal craniotomy+ Supraorbital osteotomy+ Zygomatic osteotomy+ Anterior clinoidectomy+ Para-cavernous maneuvers
Type III 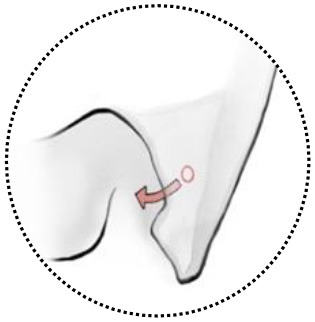	Medial surface of ACP (above the distal dural ring)	Grow medially following the dural ring, diaphragm sellae.	Frontal-temporal craniotomy + Supraorbital osteotomy+ Anterior clinoidectomy + Cutting distal dural ring +/– Drill tuberculum sellae+/– Unroof the optic canal
Type IV 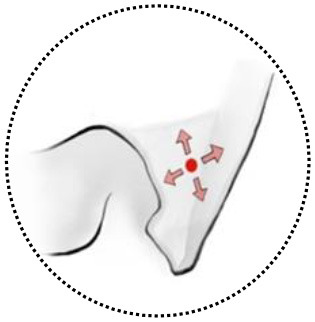	Difficult to be identified	Grow into multiple sellar and parasellar spaces, encasing the surrounding structures.	All the above surgical techniques. Carotid artery control is advocated. The patient may need a surgical plan with possible intentional partial resection to preserve important structures.

*ACP, Anterior Clinoid Process; +, with; +/−, with/without*.

Type I ACMs originated from the meninges that cover the superior surface (area I) of the ACP, extended superiorly and laterally to the suprasellar space (Figures 2A–C). On the coronal view, the epicenter of the tumor laid superior and lateral to the clinoidal ICA and optic nerve. They may extend medially following the falciform ligament into the optic canal, resulting in vision decline. The branches of the middle cerebral artery (MCA) may be pushed superiorly and posteriorly and sometimes were even wrapped inside the tumor. For type I ACMs, standard frontal–temporal craniotomy offered enough surgical exposure; complete anterior clinoidectomy was not a must, and the extent of clinoidectomy could be tailored intradurally.

Type II ACMs were clinoidal-cavernous meningiomas, which were further divided into two subgroups (Figures 2D–I). Type IIa ACMs (Figures 2D–F) originated from the meninges that covered the lateral surface of ACP (area II), extended along the lateral wall of the cavernous sinus. Since the lateral wall of the cavernous sinus consisted of multilayers of the meningeal dura, tumors usually did not transgress this layer, so the cavernous sinus was compressed rather than invaded. The epicenter of these tumors laid laterally to the clinoidal ICA and the transcavernous cranial nerves. During surgery, a zygomatic osteotomy was needed in addition to frontal–temporal craniotomy in order to provide a “low-enough” surgical trajectory. Complete clinoidectomy was not mandatory, and the extent of bony removal could be tailored based on intraoperative findings.

Type IIb ACMs (Figures 2G–I) originated from the tip of the clinoid process (area III); they not only extended laterally to the middle cranial fossa like type IIa ACMs but also into the cavernous sinus and wrapped the neurovascular structures inside the sinus following the meninges that cover the roof of the cavernous sinus and the sleeves of the oculomotor and trochlear nerves, forming a “weak point” of the cavernous sinus. The coronal view of MRI was used to differentiate between type IIa and IIb ACMs. In type IIb ACMs, cranial nerve insufficiency was commonly seen. When performing the craniotomy, frontal–temporal craniotomy with complete clinoidectomy was essential for adequate exposure. The para-cavernous maneuvers need to be applied to facilitate tumor removal inside of the cavernous sinus.

Type III ACMs (Figures 2J–L) originate from the meninges that cover the medial wall of the ACP (area IV). The tumor extended medially to the diaphragm sellae, warped the supra-clinoidal segment of the ICA, pushed the optic nerve from above and/or below, and then extend into the sellar. On the coronal MRI, the epicenter of the tumor laid superior and medial to the ICA; the pituitary stalk was often pushed to the contralateral side. During surgery, complete anterior clinoidectomy was needed for adequate exposure, and the frontal–temporal craniotomy often required to be expanded with a supraorbital osteotomy to get a wider surgical angle. Drilling the tuberculum sellae was sometimes essential for removing the tumor inside the sellae.

Type IV ACMs (Figures 2M–O) were large tumors that extended to multiple sellar and parasellar spaces, thus were not included in types I–III ACMs. For type IV tumors, preoperative angiography was strongly warranted, and tumor-feeding vessels were embolized to decrease the risk during surgery. An orbital-zygomatic craniotomy with complete anterior clinoidectomy was applied for broad exposure. Much attention and energy were needed when dissecting the vessels that were encased by the tumor. Sometimes, a subtotal resection was done on purpose to minimize the surgical morbidity and preserve the functional outcome.

### Surgical Outcomes of ACMs

A total of 2,654 patients with intracranial meningiomas were surgically treated from 1998 to 2019, while 131 patients were confirmed to be ACMs. According to the previously mentioned classification, 55 cases (42.0%), 26 cases (19.8%), 12 cases (9.2%), 22 cases (16.8%), and 16 cases (12.2%) were classified as types I, IIa, IIb, III, and IV, respectively. Their clinical and pathological features are summarized in Table 2. Type III and IV ACMs had the highest chance for presenting with vision decrease, followed by type I ACMs. Headache was mostly presented in 94% of type IV ACMs and 42% of type IIa ACMs.

**Table 2:** Clinicopathological characteristics of anterior clinoidal meningioma patients.

**Characteristics**	**All patients (*n* = 131)**	**Type I ACMs (*n* = 55)**	**Type IIa ACMs (*n* = 26)**	**Type IIb ACMs (*n* = 12)**	**Type III ACMs (*n* = 22)**	**Type IV ACMs (*n* = 16)**
**Age (years)**						
Range	27–76	33–73	27–76	27–73	33–60	38–64
Mean ± SD	53.66 ± 10.82	55.95 ± 9.68	57.31 ± 12.77	55.83 ± 12.04	46.77 ± 8.42	47.75 ± 6.78
**Gender**						
Male	45 (34.4%)	22 (40.0%)	8 (30.8%)	6 (50.0%)	8 (36.4%)	1 (6.3%)
Female	86 (65.6%)	33 (60.0%)	18 (69.2%)	6 (50.0%)	14 (63.6%)	15 (93.7%)
**Presenting symptoms**						
Vision decrease	57 (43.5%)	25 (45.5%)	1 (3.8%)	3 (25.0%)	16 (72.7%)	12 (75.0%)
Headache	45 (34.4%)	12 (21.8%)	11 (42.3%)	4 (33.3%)	3 (13.7%)	15 (93.7%)
Dizziness	32 (24.4%)	11 (20.0%)	8 (30.8%)	4 (33.3%)	3 (13.7%)	6 (37.5%)
Incidental finding	11 (8.4%)	5 (9.1%)	3 (11.5%)	1 (8.3%)	2 (9.1%)	0 (0%)
Seizure	7 (5.3%)	0 (0%)	3 (11.5%)	3 (25.0%)	0 (0%)	1 (6.2%)
Limb weakness	6 (4.6%)	1 (1.8%)	2 (7.7%)	0 (0%)	0 (0%)	3 (18.8%)
Diplopia	4 (3.1%)	0 (0%)	0 (0%)	2 (16.7%)	0 (0%)	2 (12.5%)
Ptosis	3 (2.3%)	0 (0%)	0 (0%)	1 (8.3%)	0 (0%)	2 (12.5%)
**Tumor diameter**						
≥3 cm	108 (82.4%)	40 (72.7%)	23 (88.5%)	12 (100%)	17 (77.3%)	16 (100%)
<3 cm	23 (17.6%)	15 (27.3%)	3 (11.5%)	0 (0%)	5 (22.7%)	0 (0%)
**Resection degree (simpson)**						
Grade 1–2	88 (67.2%)	47 (85.5%)	24 (92.3%)	0 (0%)	12 (54.5%)	5 (31.3%)
Grade 3–4	43 (32.8%)	8 (14.5%)	2 (7.7%)	12 (100%)	10 (45.5%)	11 (68.7%)
**Tumor grades**						
WHO grade I	120 (91.6%)	51 (92.7%)	25 (96.2%)	11 (91.7%)	20 (90.9%)	13 (81.3%)
WHO grade II	10 (7.6%)	4 (7.3%)	1 (3.8%)	1 (8.3%)	2 (9.1%)	2 (12.5%)
WHO grade III	1 (0.8%)	0 (0%)	0 (0%)	0 (0%)	0 (0%)	1 (6.2%)
**Ki-67 index**	*n* = 90	*n* = 37	*n* = 19	*n* = 11	*n* = 17	*n* = 6
<5%	73 (81.1%)	32 (86.5%)	18 (94.7%)	8 (72.7%)	13 (76.5%)	2 (33.3%)
5–10%	15 (16.7%)	5 (13.5%)	1 (5.3%)	2 (18.2%)	4 (23.5%)	3 (50%)
>10%	2 (2.2%)	0 (0%)	0 (0%)	1 (10.1%)	0 (0%)	1 (16.7%)

Nearly 70% of patients had Simpson grade I and 2 resections (88/131), while the rest (43/131, 32.9%) got Simpson grade 3–4 resection. Types IIa and I had the highest chance of total resection (92.3 and 85.4%, respectively), followed by type III (54.5%) and type IV (31.3%), while type IIb showed no gross total resection in our case series. Univariate and multivariate analyses revealed ACM classification and tumor size (<3 cm) to be independent risk factors for achieving more extensive resection (*p* = 0.024 and *p* = 0.025, respectively, Table 3).

**Table 3:** Univariate and multivariate analysis for factors associated with extent of resection of ACMs.

**Factors**	**Univariate analysis**		**Multivariate analysis**		
	**OR**	***P*-value**	**OR**	***P*-value**	**95% CI**
Age (years)		0.326		0.729	
<40	1.000	1.000	1.348	0.774	0.175-9.366
40–60	0.552	0.171	0.735	0.639	0.202-2.671
>60	Reference	Reference		Reference	Reference
Type of ACMs		<0.001^*^		0.024^*^	
I	12.925	<0.001^*^	4.769	0.065	0.909–25.017
IIa	26.400	<0.001^*^	13.314	0.014	1.684–105.270
IIb	0.000	0.999	0.000	0.999	0
III	2.640	0.159	0.714	0.726	0.108–4.705
IV	Reference	Reference	Reference	Reference	Reference
Vision decrease (yes vs. no)	0.475	0.049^*^	0.800	0.703	0.254–2.517
Headache (yes vs. no)	0.456	0.042^*^	0.419	0.207	0.108–1.618
Dizziness (yes vs. no)	1.641	0.281	1.101	0.883	0.306–3.959
Tumor diameter (<3 cm vs. ≥3 cm)	0.071	0.011^*^	0.075	0.025^*^	0.008–0.724
Gender (female vs. male)	0.966	0.929	0.784	0.671	0.2552.408

* *Statistically significant*.

**Figure 3:**
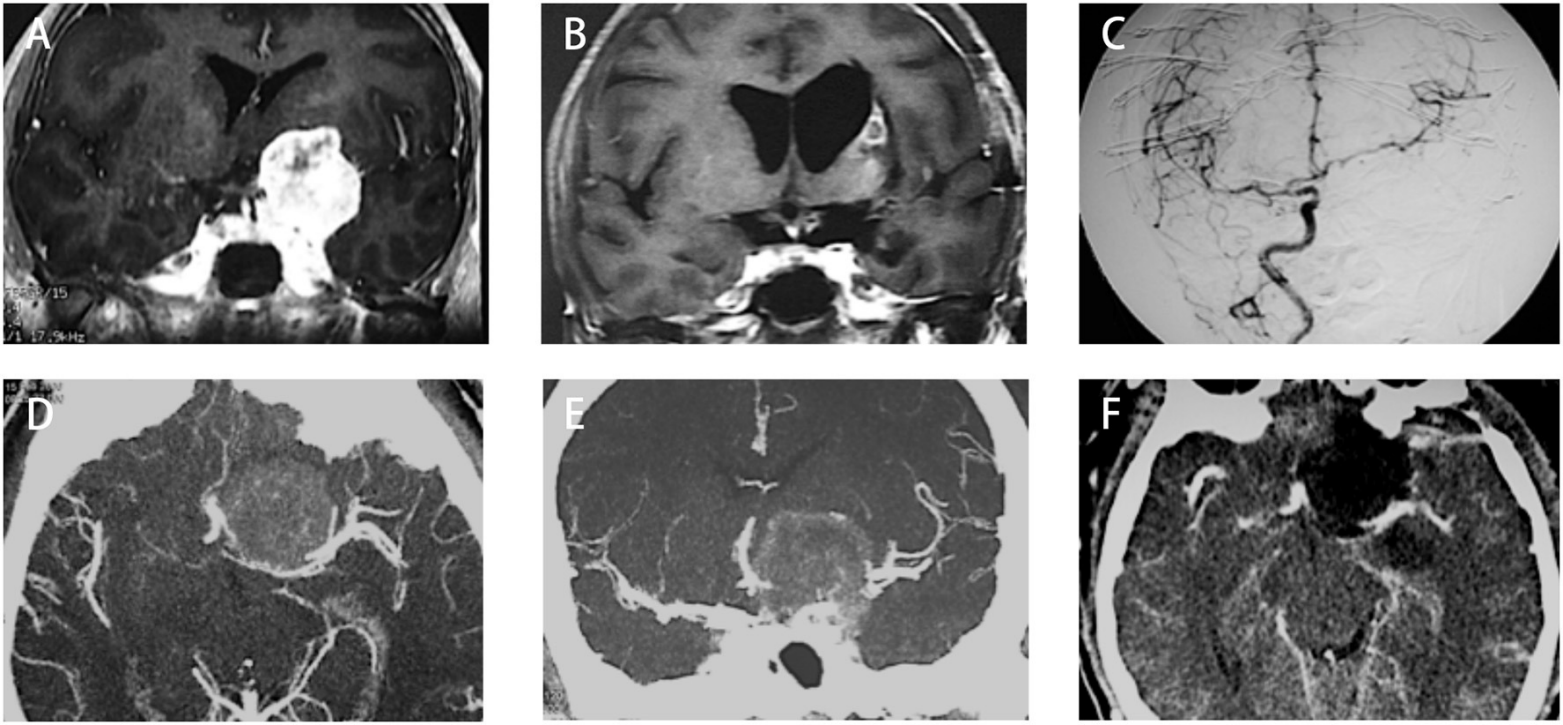
Two cases with anterior clinoidal meningiomas (ACMs) that experienced intraoperative rupture of internal carotid artery (ICA). **(A)** Preoperative coronal MRI showed a type I ACM, which involved the supraclinoidal part of the left ICA. **(B)** Postoperative coronal MRI showed gross-total resection of tumor, with a small infarction near the left side lateral ventricle, indicating the occlusion of perforators supplying the head of the caudle nuclei. **(C)** Postoperative angiography showed complete occlusion of the left ICA. **(D,E)** Preoperative CT angiography showed a type III ACM, which engulfs the bifurcation of the left ICA. **(F)** Postoperative CT angiography showed patent branches of the ICA with gross total removal of the tumor.

We had one perioperative death because of severe meningitis after surgery. Two cases of ICA rupture were found during operation when the chief operator was trying to dissect the tumor from the ICA with no arachnoid plane in between. Both cases were treated using compression and surgical glue repairing. On postoperative imaging, one patient (type I, Figures 3A–C) had total occlusion of the ipsilateral ICA but showed no neurological deficits. Another patient (type III, Figures 3D–F) showed a patent lumen of the ICA with no signs of a pseudoaneurysm. Both patients reported good status with no signs of recurrence (9 and 12 years after surgery, respectively).

For the patients with preoperative vision decrease, 52.6% got improved after surgery, while 42.1% were unchanged, and 5.3% had deterioration. A newly onset of vision loss after surgery was found in five cases (one case with type I, one case with type III, and the other three cases with type IV) of which two patients became better after treatment in the hyperbaric oxygen cabin. Oculomotor nerve dysfunction was found in three cases preoperatively and remained unchanged after surgery. A newly onset of oculomotor palsy was found in 18 cases (2 cases with type I, 3 cases with type IIa, 5 cases with type IIb, 1 case with type III, and the other 7 cases with type IV), of which 12 turned out to be temporary and had recovery gradually during follow-up.

The postoperative pathological study revealed most of the ACMs in our series to be WHO grade I (91.6%), with 7.6% diagnosed as WHO grade II and only one case confirmed to be WHO grade III. Of 90 tumor samples in which Ki-67 was tested, 81.1% showed <5% intensity, 16.7% showed intensity between 5 and 10%, and the rest 2.2% showed >10%.

Adjuvant radiotherapy was recommended to the patients with incomplete resection and/or high WHO grades. Of the 131 patients, 114 had the follow-up, with 13 cases of recurrent or regrowth (1 case of type I, 0 cases of type IIa, 2 cases of type IIb, 4 cases of type III, and 6 cases of type IV) and one death caused by the recurrent tumor (type IV, WHO grade III). Type III and IV ACMs had a higher chance of recurrence/regrowth, since most of these types of tumors did not get total resection. The remaining tumor in the cavernous sinus (type IIb) seemed relatively stable after radiotherapy; only 2 out of 12 cases had regrowth in the follow-up period.

## Discussion

### Meningeal Architecture-Based Classification of ACMs

Meningiomas originate from the meningothelial cells at the dural–arachnoid junction (12) and tend to grow along with the meningeal layer of the dura (13, 14), so the meningeal architecture plays an essential role in determining the potential extending pathway and selecting appropriate surgical maneuvers for resection ACMs (15). In the present study, we used a novel morphological technology, the epoxy sheet plastination in combination with the confocal microscopy, to identify and trace the meningeal layers around the ACP and reveal their precise relationship with surrounding structures, e.g., the ICA, cranial nerves, and cavernous sinus (Figure 1F). The meningeal fibrous configuration of the ACP, optic canal, and cavernous sinus reported in this study and our previous studies provide an anatomical base for our new classification of the ACMs (16–18).

Several classification systems of ACMs were established in the past decades. AI-Mefty developed a grading system that divided the ACMs into group I for tumors arising proximal to the end of the carotid cistern, group II for tumors arising above the segment of the carotid invested in the carotid cistern, and group III for tumors originated from the optic foramen (1). Pamir modified this system by adding the diameter of tumors (<2, 2–4, and >4 cm) for each group (19). Goel invented a 2–10 scoring system based on the extent of visual impairment, size of the tumor, and tumor relationship with the ICA (20). Nakamura et al. divided the ACMs into only two groups: group 1 for tumors not invading cavernous sinus and group 2 for tumor involving the cavernous sinus (21). Nanda et al. established another grading system with total scores ranging from 1 to 10, then divide the patients into group 1 with scores <5 and group 2 with scores >5 (22).

There are some limitations to these classifications. First, a complicated scoring system is neither user-friendly nor easy to follow. Second, these classifications did not demonstrate the underlying relationship among the tumor, ACP, and surrounding structures that were linked by the meningeal anatomy. The classification system that we proposed herein provided another angle of view to the ACMs, in addition to the current knowledge of how ACMs originated, extended, presented, and resected. Different groups of ACMs may have different clinical presentations, requiring various surgical maneuvers and leading to different surgical outcomes. With the information provided by the classification, in addition to the diagnostic radiological findings, the surgeons can have a better preoperative estimation and a better prediction of the surgical outcomes for one specific case of ACM.

### Surgical Approach for Removing ACMs

Many authors reported different approaches for removing ACMs, like lateral supraorbital approach (23), supraorbital keyhole approach (24), standard or extended pterional approach (4, 25–27), frontal lateral approach (21), orbital-zygomatic approach (28–32), etc. Table 4 summarized different surgical approaches and techniques as well as the outcomes that were published in the past decades. For ACMs, we believe that no universal approach could fit all the needs, nor do we advocate a routine clinoidectomy or unroofing the optic canal (34). Generally, a frontal–temporal craniotomy offers good anterior-lateral working trajectory and could be applied in most type I tumors, while other skull base techniques, like zygomatic osteotomy, orbital osteotomy, unroofing the optic canal, drilling the tuberculum sellae, etc., could be used either single or combined based on the tumor characteristics. The endoscopic endonasal route offers a different working angle, but current reports only limited in small size ACMs (35, 36).

**Table 4:** Literature review of recently published papers of anterior clinoidal meningiomas.

**References**	**No. of patients**	**Mean/Median follow-up (months)**	**Total resection (%)**	**Post-operative visual function improvement (%)**	**Recurrence (%)**	**Surgical approach**	**Pathological findings**
Al-Mefty. (1)	24	57	83.3	25	12.5	Pterional/subfrontal/orbitocranial	NA
Kleinpeter Böck (5)	31	NA	77.4	NA	NA	NA	NA
Risi et al. (26)	34	22.8	58.8	32	21	Extended pterional	NA
Puzzilli et al. (25)	33	53.7	54.5	33.3	15.2	Pterional	WHO I, *n* = 29 WHO II, *n* = 4
Goel et al. (20)	60	26	70.0	69.1	1.6	Basal frontotemporal/orbitozygomatic	NA
Nakamura et al. (21)	108	79	42.6	46.7	20.3	Pterional/frontolateral	WHO I, *n* = 105 WHO II, *n* = 2 WHO III, *n* = 1
Russell et al. (27)	35	153.6	68.6	63	9	Pterional	NA
Cui et al. (28)	26	22.3	61.5	61.5	0	Orbitozygomatic	NA
Pamir et al. (19)	43	39	90.7	84.6	11.7	Pterional	WHO I, *n* = 42 WHO II, *n* = 1
Sade and Lee (2)	52	NA	71.2	77	NA	Pterional + posterolateral orbitotomy + clinoidectomy	NA
Bassiouni et al. (33)	106	83	57.5	45.6	25.3	Pterional + optic nerve decompression + subdural clinoidectomy	WHO I, *n* = 102 WHO II, *n* = 2 WHO III, *n* = 1
Romani et al. (23)	73	36	78.1	28.2	4.1	Lateral supraorbital	WHO I, *n* = 66 WHO II, *n* = 7
Nagata et al. (29)	23	49.2	39.1	43.5	4.3	Pterional/orbitozygomatic	NA
Attia et al. (30)	22	56	59.1	66.7	13.6	Pterional/frontoorbital/orbitozygomatic	WHO I, *n* = 19 WHO II, *n* = 3
Czernicki et al. (31)	30	83	63.3	43.8	36.8	Fronto-orbitozygomatic	WHO I, *n* = 30
Sughrue et al. (34)	29	90	20.7	17.2	6.9	Frontotemporal/orbitozygomatic	WHO I, *n* = 27 WHO II, *n* = 2
Nanda et al. (22)	36	33	75.0	28	11.1	Pterional/orbitozygomatic	WHO I, *n* = 36
Kim et al. (32)	59	54.1	64.4	NA	18.6	Orbitocranial or orbitozygomatic/extended pterional/subfrontal	NA
*Present study*	131	76	67.2	52.6	11.4	Frontotemporal craniotomy with individualized skull base techniques	WHO I, *n* = 120 WHO II, *n* = 10 WHO III, *n* = 1

*No., Number; NA, Not Applicable*.

### Surgical Outcomes of ACMs

**Figure 4:**
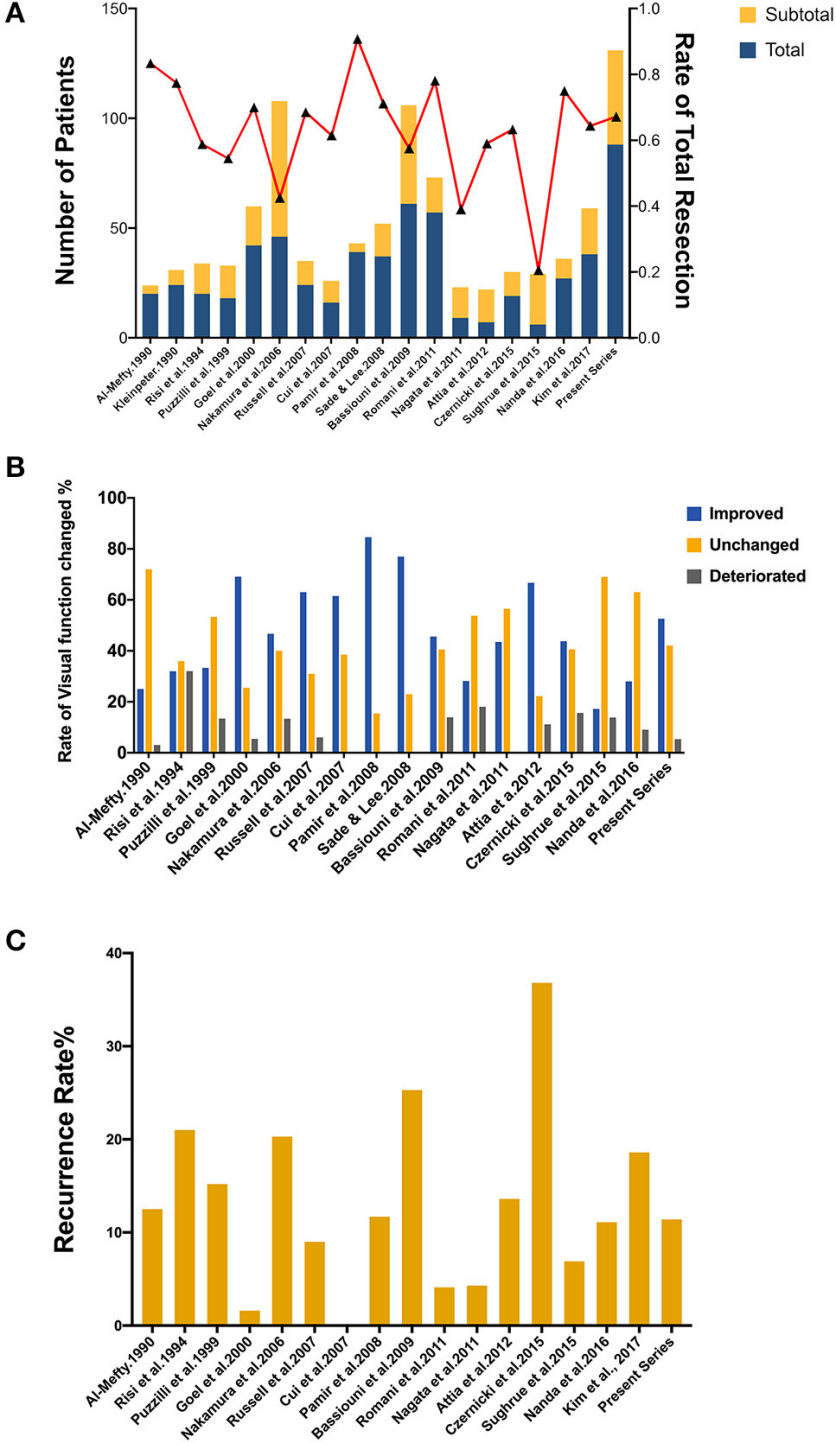
Summary of literature on the extent of resection, visual function and tumor progression of ACMs. **(A)** A total of 18 studies (excluding the present study) reported the extent of resection of ACMs, ranging from 20.7–90.7%. **(B)** The rate of visual function improvement among 16 studies. 37.5% of studies (6/16) revealed improvements in visual function in more than 50% of patients. **(C)** The progression/recurrence rate varies from 0–36.8% in 16 studies.

Up to date, many studies have reported the surgical treatment of ACM, but large series that include more than 100 patients with a long follow-up period were only seen in two series (21, 33). The rates of total resection ranging from 42.6 to 90.7%, with a varied chance of visual function improvement and tumor recurrence (Figure 4). In the present study, the total resection rate was 67%, with the visual function improved in 52.6% of patients and recurrent in 11.4% patients during the follow-up.

Arachnoid plane, as many authors have mentioned before, is the crucial factor for resectability. When an arachnoid plane is available, even when the vessels are encased by the tumor, experienced surgeons may still be able to dissect the vessels out of the tumor with microsurgical skills. Lack of arachnoid plane may cause firm adhesion between tumor and critical neurovascular structures, leading to a higher chance of complication during dissection.

Our anatomical study revealed a very close relationship between the distal dural ring (DDR) to ICA adventitia. Thus, type III ACMs can extend directly from the DDR to the surface of ICA, without leaving any arachnoid plane. Our finding was consistent with another histological study, which also found that the meningeal DDR eventually fuses with the adventitia of the ICA (37).

The genomic invasiveness might be another reason; some recently published studies found that even WHO grade I meningioma could show high invasive molecular behavior-like high-grade meningiomas, if they harbor specific genetic background. The tumor can cause severe edema (which indicated disruption of the arachnoid and pia), invasion to the brain, and easy recurrence, et al. (38, 39). This may explain the intraoperative ICA rupture case (type I, WHO grade I, Figure 3A). Future studies that focus on these low-grade but invasive meningiomas may help identify this subgroup of tumors before surgery.

### ACMs Invading Cavernous Sinus

Cavernous sinus invasion rate of ACMs ranges from 0 to 63.9% (20, 21, 26, 40). The various ranges may be due to mixing tumors that compress the lateral wall with the tumors that truly invade into the cavernous sinus. In our study, we found that the “true” cavernous sinus invasion rate was 17.6%.

The meningeal anatomy of the lateral wall for the cavernous sinus has been extensively studied. The two-layer model was described by Umansky et al. (41), then widely accepted both anatomically and surgically (42–44). Janjua found that there is another intermediate layer between these two layers, which showed a distinct fiber direction of the other two layers (9). We reported that the lateral wall of the cavernous sinus is formed by the multiple-layered meningeal, which forms a relatively tight barrier for meningiomas to transgress the lateral wall, enters into the cavernous sinus, and engulfs the internal carotid artery (17).

The roof of the cavernous sinus is continuous with the superficial layer of the lateral cavernous wall, is relatively “weak,” and can be pierced by the tumor. Via the roof, the meningioma can invade into the cavernous sinus, wrap the carotid artery and cranial nerves, making a total ACM resection much more complicated and riskier, which warrants a more conservative surgical therapeutic option. Adjuvant radiotherapy is strongly recommended for residual tumors in the cavernous sinus in order to prevent an early recurrence. Understanding of above-mentioned features helps the surgeon set up appropriate surgical goals for type IIa and IIb ACMs “involving” in the cavernous sinus.

### Limitations

Our study has some limitations. First, because of the retrospective nature of this study, the effects or outcomes of applying either this classification or other previously reported schemes in ACM patients could not be evaluated. It still needs to be assessed by future perspective studies. We added that in the limits of our current study. Second, surgical outcomes are highly dependent on the operator. In the current study, the surgeries were performed by several senior surgeons; although all of them are experienced operators, their surgical techniques and principle are not precisely the same, and this type of bias cannot be adjusted in this study. Third, although we used group discussion to determine which type a specific case should be assign to, the process still had a subjective nature. In addition, the pathological types and genomic background of each tumor were not included because of insufficient data. Future perspective studies are warranted to confirm the findings of the current study and evaluate the effect of applying this classification to the surgical outcomes.

## Conclusion

The meningeal architecture around the ACP may guide and determine the origin and extension of ACMs. The classification based on the meningeal architecture helps to understand surgical anatomy as well as predicting surgical outcomes.

## Data Availability Statement

The datasets generated for this study are available on request to the corresponding author.

## Ethics Statement

The cadaveric study was reviewed and approved by the Human Research Ethics Committee in University of Otago (H18/027), the clinical study was reviewed and approved by the IRB of Changzheng Hospital, 2018SL020. The patients/participants provided their written informed consent to participate in this study.

## Author's Note

Part of this work was accepted and presented in an oral presentation at the Special World Congress, World Federation of Neurosurgical Societies, Beijing, China, on Sep 10, 2019.

## Author Contributions

TX, YY, YL, MZ, and JC conceived or designed the work. TX, YY, ZG, LJ, HW, WL, LC, and HS acquired, analyzed, or interpreted data. TX, YY, and ZG drafted the paper. AE, YL, MZ, and JC revised it critically for important intellectual content. MZ and JC agreed to be accountable for all aspects of the work in ensuring that questions related to the accuracy or integrity of any part of the work are appropriately investigated and resolved. All authors provided approval for publication of the content.

## Conflict of Interest

The authors declare that the research was conducted in the absence of any commercial or financial relationships that could be construed as a potential conflict of interest.
